# Daily Temperatures and Child Hospital Admissions in Aotearoa New Zealand: Case Time Series Analysis

**DOI:** 10.3390/ijerph21091236

**Published:** 2024-09-19

**Authors:** Hakkan Lai, Jeong Eun Lee, Luke J. Harrington, Annabel Ahuriri-Driscoll, Christina Newport, Annette Bolton, Claire Salter, Susan Morton, Alistair Woodward, Simon Hales

**Affiliations:** 1 Section of Epidemiology and Biostatistics, University of Auckland, Auckland 1023, New Zealand; a.woodward@auckland.ac.nz; 2 Department of Statistics, University of Auckland, Auckland 1010, New Zealand; kate.lee@auckland.ac.nz; 3 Te Aka Mātuatua School of Science, University of Waikato, Hamilton 3216, New Zealand; 4 Te Kura Mātai Hauora School of Health Sciences, University of Canterbury, Christchurch 8041, New Zealand; annabel.ahuriri-driscoll@canterbury.ac.nz; 5 School of Population Health, University of Auckland, Auckland 1023, New Zealand; c.newport@auckland.ac.nz; 6 Institute of Environmental Science and Research, Christchurch 8041, New Zealand; annette.bolton@esr.cri.nz (A.B.); claire.salter2@cdhb.health.nz (C.S.); 7 Research Institute for Innovative Solutions for Well-being and Health (INSIGHT), Faculty of Health, University of Technology Sydney, Sydney, NSW 2007, Australia; susan.morton@uts.edu.au; 8 Department of Public Health, University of Otago, Wellington 6011, New Zealand

**Keywords:** climate change, heat stress, child morbidity, hospitalisation, deprivation, ethnicity

## Abstract

The influence of global climate change on temperature-related health outcomes among vulnerable populations, particularly young children, is underexplored. Using a case time series design, we analysed 647,000 hospital admissions of children aged under five years old in New Zealand, born between 2000 and 2019. We explored the relationship between daily maximum temperatures and hospital admissions across 2139 statistical areas. We used quasi-Poisson distributed lag non-linear models to account for the delayed effects of temperature over a 0–21-day window. We identified broad ICD code categories associated with heat before combining these for the main analyses. We conducted stratified analyses by ethnicity, sex, and residency, and tested for interactions with long-term temperature, socioeconomic position, and housing tenure. We found J-shaped temperature–response curves with increased risks of hospital admission above 24.1 °C, with greater sensitivity among Māori, Pacific, and Asian compared to European children. Spatial–temporal analysis from 2013–2019 showed rising attributable fractions (AFs) of admissions associated with increasing temperatures, especially in eastern coastal and densely populated areas. Interactive maps were created to allow policymakers to prioritise interventions. Findings emphasize the need for child-specific and location-specific climate change adaptation policies, particularly for socioeconomically disadvantaged groups.

## 1. Introduction

Global climate change has been described as one of this century’s greatest threats to public health [1]. However, most countries have been sluggish in addressing this issue, risking severe and potentially irreversible health consequences [2]. Although the 2015 Paris Agreement aims to limit global average temperature rise to well below 2 °C, present international commitments are insufficient to achieve this goal [3]. Children are physiologically and metabolically more sensitive to unfavourable climate than adults [4] and bear an estimated 88% of the burden of climate-related diseases, primarily because their young age leads to a greater loss of healthy life years [5]. One reason for this increased vulnerability is that children have a higher surface area-to-body mass ratio compared to adults, which makes them more susceptible to heat and dehydration [6]. Additionally, their developing immune systems and incomplete physiologic responses make them more vulnerable to extreme weather conditions [6]. Furthermore, children’s exposure to pollutants and allergens can be exacerbated by climate change, leading to respiratory diseases such as asthma [7].

One of the most rapidly worsening climate hazards is the increasing frequency of extreme heat events [8]. A recent study found that as much as a third of heat-related deaths in the past 20 years can be attributed to human-induced climate change [9]. With global warming of 2 °C, locally extreme hot days (defined as having a 0.2% chance of occurrence in any year in a pre-industrial climate) are expected to occur 14 times more often, and are projected to cause severe and widespread impacts on heat-related morbidity and mortality [10]. Enhancing our understanding of young children’s susceptibility to heat will help policymakers and stakeholders make more informed decisions [11,12]. Year-on-year increases in global temperatures point to the need for concerted efforts to safeguard public health and well-being, particularly for future generations, who are projected to suffer more frequently than the current generation.

Very few previous studies have reported associations between children’s exposure to high temperatures and daily hospital admissions [4,13,14]. Admissions of young children to hospital disrupt preschool and family activities, and may signal enduring health issues that persist into adulthood [15]. The impact of temperature on health typically follows a “U-shaped” pattern, where risks of illness increase as temperatures deviate from an optimal range [16,17]. Due to acclimatisation and social adaptation, this optimal range varies from country to country, and could be lower in high-latitude countries, such as Canada, Sweden, and the United Kingdom [18].

In Aotearoa New Zealand (NZ), local climates vary considerably, ranging from subtropical to temperate in the north, while much colder inland alpine and subantarctic conditions apply in the south. Similarly, the ethnic composition of the population also varies significantly from north to south, with higher proportions of Māori and Pacific Island communities in the north, and a greater predominance of European communities in the south. Furthermore, childhood hospital admission rates differ by ethnicity: admission rates are higher for Māori and Pacific Island children than for other ethnic groups. For instance, between 2017 and 2021, Māori and Pacific children’s hospitalisation rates for respiratory conditions were 2 and 2.5 times higher than those of children of European or other ethnicities [19]. Ensuring environmental equity is crucial to maintaining a fair distribution of resources to protect against environmental impacts, including heat-related health effects. Assessing heat–health relationships by spatial, socio-demographic, housing, and environmental factors can help target interventions where they are most needed. This is particularly important in NZ, where historical and ongoing disparities have resulted in inequitable exposure and vulnerability to environmental hazards by ethnicity [20].

We aimed to quantify short-term associations between local ambient temperatures and childhood hospitalisation; assess whether these associations varied by individual-level and area-level factors; and estimate temporal and spatial patterns of related health impacts.

## 2. Materials and Methods

Analyses were conducted in the secure data laboratory of the Integrated Data Infrastructure (IDI), a database managed by Statistics New Zealand that integrates de-identified data from the government and various sectors. This facility supports statistical analyses by tracking individuals’ life events over time. We included 647,000 hospital admissions from children born between 1 January 2000 and 31 December 2019, who were admitted to public hospitals during the same period and were under five years old at the time of admission (hereafter “child admissions”). We excluded hospital admissions within the first five days post-birth to eliminate early readmissions unrelated to heat [21]. We accounted for same-day multiple admissions by retaining only the last event per day, addressing same-day referrals between hospitals and leveraging the diagnostic insights from the final hospital visited. Hospital records provided by the Ministry of Health included the date of hospital admission, date of birth, and diagnosis codes according to the International Classification of Diseases, tenth revision (ICD-10). We analysed the principal and the first two secondary causes of hospital admission, i.e., the first three causes, that were potentially associated with ambient heat exposure. Note that New Zealand adopted the use of the ICD-10 in 1999, ahead of several other countries, including the United States, France, Italy, South Africa, and China.

Our selection of ICD code(s) relevant to heat-related child admissions was done in three stages: (i) We initially combined ICD codes broadly derived from general population studies to define the model parameters. (ii) We stratified daily admission counts by broad categories of selected ICD codes to assess statistical associations and biological plausibility for each category. (iii) We then refined the ICD codes within each category and combined all categories that showed statistical associations with heat, creating our definition of heat-related causes for child admissions. This definition was subsequently used to aggregate daily admission counts, which were applied to the main statistical model for analyses.

Based on child and parental records in the IDI, we linked hospital admission records to the most contemporaneous meshblock of residence (meshblocks are the smallest available geographical units in NZ, containing approximately 100 people on average, N = 46,637). Daily total admission counts were summed within statistical areas (SA2, N = 2139), medium-sized geographic areas. We chose to model the full range of temperature effects, although our primary interest was in heat effects. Initial exploratory models used either daily minimum, mean, or maximum temperature or apparent temperature from land station time series as the main explanatory variable of interest. The daily maximum temperature gave consistent results and was used in all subsequent models. Daily maximum temperatures from 2000 to 2019 were based on gridded data from the Virtual Climate Station Network (VCSN) at a 5 km resolution [22]. Meshblock-level temperature data were aggregated to the SA2 level based on the maximum within each SA2.

We used the case time series design to examine the effects of temperature on admissions across small areas (SA2s) [23]. This is a variation of the distributed lag non-linear model (DLNM), which controls for time-invariant confounders at the SA2 level by design and for which there are well-established methods of model selection [18].

As in previous studies, we used a fixed effect quasi-Poisson DLNM, with an exposure–lag window spanning 0 to 21 days [24] to capture delayed effects of heat and cold (Supplemental Figure S1). Natural cubic splines were employed to model temperature effects, with knots placed at the 50th and 90th percentiles of daily maximum temperature (Supplemental Figure S2). The data were stratified by year, month, and location (SA2) [23]. The day of the week was included as a dummy variable. The model expression is

logE [y_*t*,*i*_] = α*_i_* + βY*_t_* + θM*_t_* + λDoW*_t_* + γB (T_*t*,*i*_, L) + µX*_i_*

where y_*t*,*i*_ is the number of admissions for date *t* in the *i*^th^ SA2 level; α*_i_* represents the SA2 level-specific effect; Y*_t_* is the year dummy variable; M*_t_* is the month dummy variable; DoW*_t_* is the day-of-week dummy variable; B(T_*t*,*i*_, L) is the bi-dimensional exposure–lag crossbasis function of temperature, and T with lag 0 to L days and α*_i_* is the fixed effect of the *i*^th^ SA2 level. X*_i_* is a scaled additional covariate associated with the SA2 level, applied in some models to test for interaction.

We estimated relative risks (RRs) of hospital admission relative to the reference temperature at which the risk of hospital admission is minimised. We fitted models stratified by individual-level factors, including ethnicity (total responses in Māori; Pacific Peoples; Asian; sole European; and Other for all other residual groups, including ethnic groups from the Middle East, Latin America, and Africa), sex (male, female), and residency (resident, non-resident). Additionally, we explored interaction effects for area-level factors, including population-weighted long-term average temperature, socioeconomic deprivation (using population-weighted average NZDep2013 scores), and home ownership (categorized by proportions of home-owning households, as detailed in Supplemental Figure S3). This approach provides an indirect estimation by testing one hypothesis at a time and separately exploring the effects at low or high levels of these factors. For cases where minimised RRs were not observed, we confined the reference temperature to within ±3 °C of the main model’s reference temperature.

We calculated the attributable fractions (AFs) in lag 0–21 days for each SA2 using a backward method [25]:

$$AF=\frac{\sum_{j=1}^{J}\left(\frac{\left({RR}_{j}-1\right)}{{RR}_{j}}n_{j}\right)}{\sum_{j=1}^{J}n_{j}}$$

where n*_j_* is the number of admissions at day *j*; *J* is the total number of days.

Due to computing capacity limitations, we calculated the AF based on the model from the last seven years of the study period (2013–2019). This was done using the main model’s reference temperature after evaluating its temporal (Supplemental Figure S4) and spatial (Supplemental Figure S5) representativeness.

We plotted temporal patterns of the AF from 2013 to 2019 alongside population-weighted metrics of temperature data. Further, we used attributable numbers (ANs), using AN = AF × n, to evaluate the impact of climate change, assuming a uniform increase across all daily maximum temperatures of up to 3 °C. Spatial patterns of AFs were illustrated on the maps for this period. We also illustrated the attributable density (AD) on the map, defined as AN per 10 km^2^.

We used SAS (Enterprise Guide version 7.1), for data management, and R (version 4.3.1), for data analyses. We obtained ethics approval (UAHPEC23785) for research and IDI approval (MAA2022-01) for using de-identified data in compliance with the confidentiality requirements.

## 3. Results

**Figure 1:**
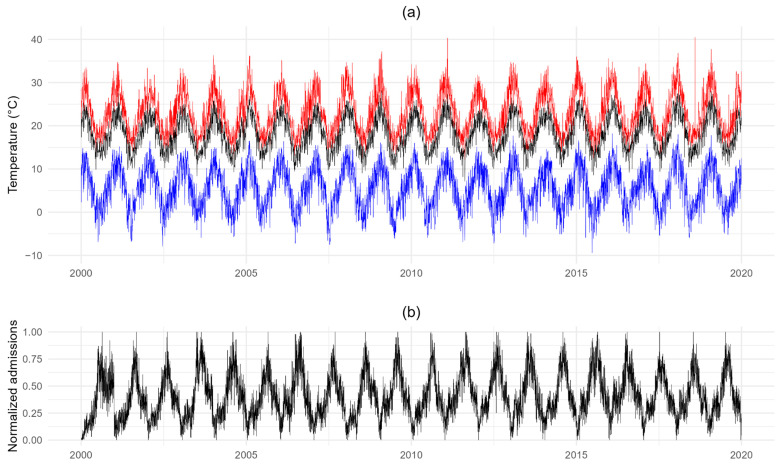
Patterns of (**a**) daily maximum temperatures and (**b**) daily child hospital admissions from 2000 to 2019. (Note: meshblock-level aggregation for maximum of daily maximum temperature (red), minimum of daily maximum temperature (blue), and population-weighted average of daily maximum temperature (black); daily child admission counts were processed by seasonal-trend decomposition based on LOESS before normalizing to each year’s value between 0 and 1).

Daily maximum temperatures at the meshblock level ranged from −9.4 °C to 40.5 °C. Figure 1 shows this temperature range over time, alongside population-weighted average values aggregated at the meshblock level. The proximity of the population-weighted average series (mean 18.1 °C, range 7.1 °C to 28.1 °C) to the maximum series suggests that most of the population resided in relatively warmer areas. Over this 20-year period, weeks 3 to 6 (from mid-January to early February) had higher weighted average values (mean > 23 °C) than the other weeks (mean < 23 °C). On the other hand, weeks 31 to 34 (from late July to late August) had higher average child hospital admission counts than the other weeks (Figure 1). Nevertheless, the average child admissions during weeks 3 to 6 were 45% of those during weeks 31 to 34. Average annual rates of heat-related child hospital admissions in this period were 11.9% among Māori, 13.4% for Pacific Peoples, 6.1% for Asian, 7.3% for sole European, and 5.5% for Other ethnic groups.

**Figure 2:**
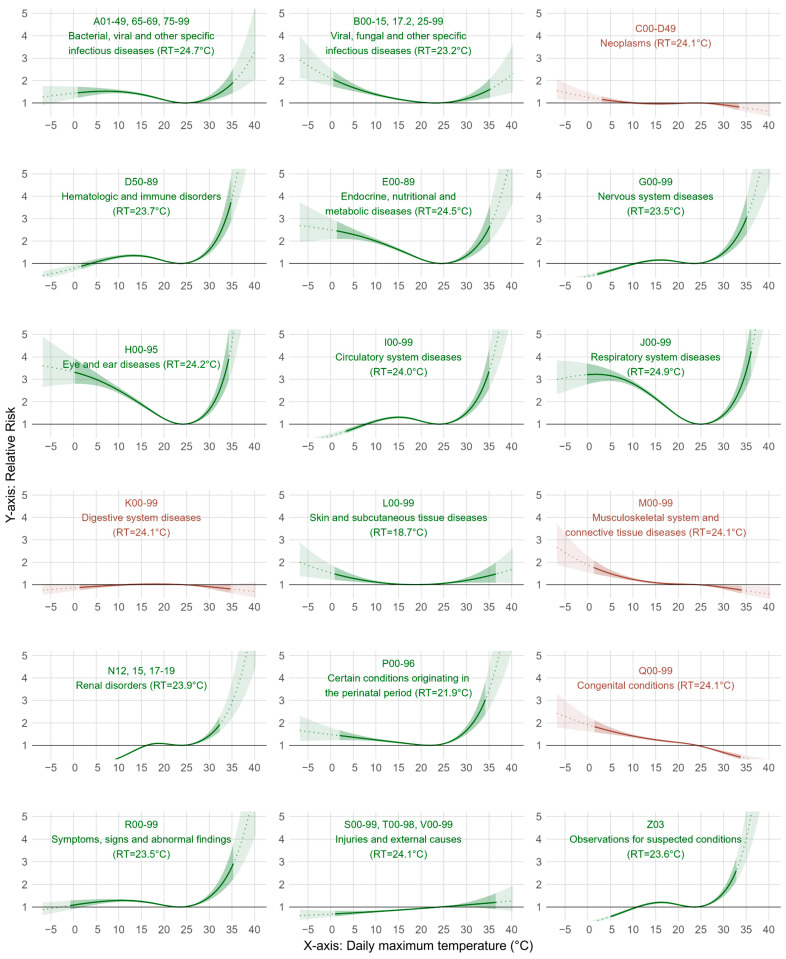
Temperature–response curves (cumulative lag 0–21 days) for child admissions in 2000–2019, stratified by ICD categories. (Note: 95% CI are shaded. Model-predicted RR and 95% CI beyond observable temperature range for admissions are in dotted line and lighter shade. For C00-D49, K00-99, M00-99, Q00-99, and S00-V99, reference temperature (RT) of 24.1 °C from the main model was used because minimal RR was not reached. Green: ICD codes selected to define heat-related causes. Brown: ICD codes not selected as part of heat-related causes).

Figure 2 presents the selected ICD codes that were used to define heat-related child admissions on cumulative lags of 0–21 days (see also Supplemental Figure S6). These included infectious diseases (A01-49, 65-69, 75-99; B00-15, 17.2, 25-99), haematologic and immune disorders (D50-89), endocrine, nutritional, and metabolic diseases (E00-89), nervous system diseases (G00-99), eye and ear diseases (H00-95), circulatory system diseases (I00-99), respiratory system diseases (J00-99), skin and subcutaneous tissue diseases (L00-99), renal disorders (N12, 15, 17-19), and conditions originating in the perinatal period (P00-96). In addition, hospital admissions related to symptoms, signs, and abnormal findings (R00-99), injuries and external causes (S00-99, T00-98, V00-99), and observations for suspected conditions (Z03) were also associated with heat effects.

**Figure 3:**
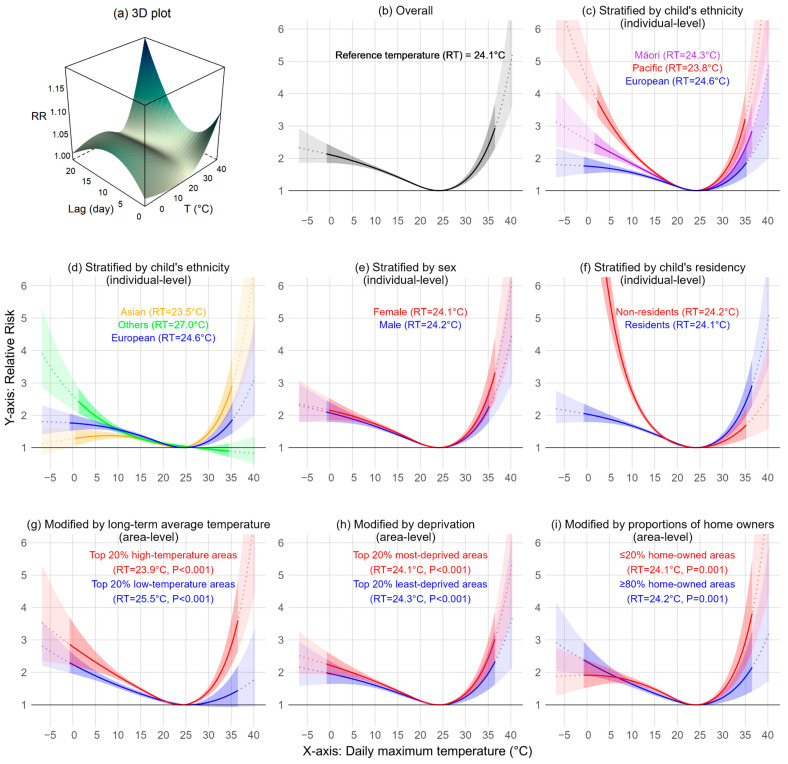
Temperature–response patterns (lag 0–21 days) for all child admissions and by sub-categories, 2000–2019. (Note: for (**b**–**i**), 95% CI are shaded, model-predicted RR and 95% CI beyond observable temperature range for admissions are in dotted line and lighter shade).

Figure 3a illustrates the association between temperature and child admissions for the above ICD codes combined, over lag 0–21 days. At 35 °C, the risk of admission was increased on day 0 (RR: 1.027 [95%CI: 1.013–1.083]) and on day 21 (1.101 [1.080–1.123]). At 5 °C, the risk of admission was increased at intermediate times, for example on day 11 (RR 1.045 [1.040–1.049]).

The following description of Figure 3 focuses on RR at 30 °C and 35 °C to reflect children’s sensitivities to heat effects at higher temperatures. Figure 3b shows a J-shaped cumulative-lag exposure–response pattern in our main model over lag 0 to 21 days. The reference temperature was 24.1 °C, with the RR increasing both above and below this point. The increase in risk was steeper at higher temperatures; RR was 1.331 [1.233–1.438] at 30 °C and 2.379 [1.950–2.902] at 35 °C.

Figure 3c,d show that when the child admission counts were stratified by ethnicity, Pacific children appeared most sensitive (RR from 1.501 [1.370–1.644] at 30 °C to 3.219 [2.550–4.063] at 35 °C), followed by Asian children (from 1.449 [1.307–1.608] to 2.825 [2.170–3.677]), Māori children (from 1.313 [1.205–1.430] to 2.314 [1.850–2.894]), and European children (from 1.201 [1.093–1.318] to 1.813 [1.417–2.319]). No significant effects of heat were found among children in the Other ethnic groups.

When stratified by sex (Figure 3e), female children appeared more sensitive to heat (from 1.379 [1.258–1.513] to 2.623 [2.068–3.326]) than male children (from 1.296 [1.188–1.415] to 2.208 [1.761–2.769]).

When stratified by residency status (Figure 3f), residents appeared more sensitive to heat (from 1.329 [1.229–1.438] to 2.370 [1.936–2.900]) than non-residents (from 1.183 [1.064–1.315] to 1.669 [1.264–2.205]).

Figure 3g shows effect modification by area-level long-term temperatures (Chi-square *p* < 0.001), indicating that children in the top 20% high-temperature areas, compared to the remainder, were sensitive to heat (from 1.421 [1.291–1.563] to 2.811 [2.194–3.602]). For children in the bottom 20% low-temperature areas, heat effects at higher temperatures were not significantly different (from 1.076 [0.949–1.221] to 1.328 [0.937–1.883]) when compared to the rest.

Figure 3h shows effect modification by area-level deprivation (*p* < 0.001), showing that children in the 20% most-deprived areas, compared to the remainder, were more sensitive to heat (from 1.345 [1.242–1.456] to 2.439 [1.987–2.994]). Conversely, children in the 20% least-deprived areas were also sensitive to heat, but the effect was weaker (from 1.243 [1.108–1.394] to 1.967 [1.460–2.649]).

Figure 3i shows the effect modification by home ownership (*p* = 0.001). Children in areas with ≤20% owner-occupied households, compared to the remainder, showed increased sensitivity to heat (from 1.432 [1.275–1.608] to 2.934 [2.174–3.959]). Similar patterns were also observed for the proportion of privately rented homes (Supplemental Figure S7), but the effect modification was not statistically significant.

**Figure 4:**
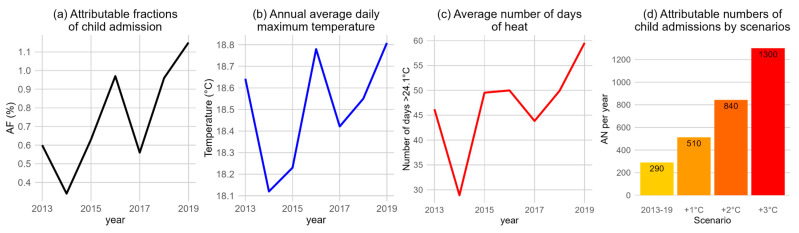
Child hospital admissions (2013–2019) attributable to heat effects >24.1 °C (cumulative lag 0–21 days): (**a**) attributable fractions (%), (**b**) annual temperature, (**c**) temperature exceedance, and (**d**) overall attributable numbers across three climate change scenarios. (Note: (**b**–**c**) were based on population-weighted average from meshblock-level data from 2013).

**Figure 5:**
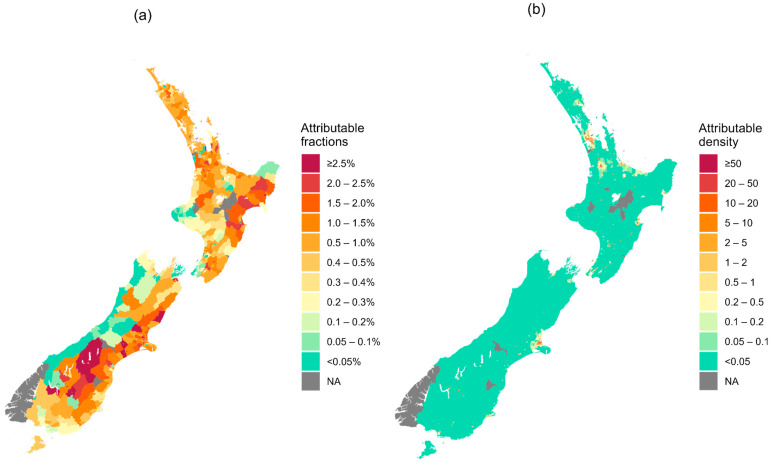
Child hospital admissions (2013–2019) due to heat effects > 24.1 °C (cumulative lag 0–21 days), (**a**) attributable fractions (%) and (**b**) attributable density (attributable number per 10 km^2^) in statistical area (SA2) level. (Note: see interactive HTML maps in Supplemental Figure S9).

Figure 4 and Figure 5 are based on analyses of the last seven years of the study period, 2013–2019.

Figure 4 shows the increasing trends of attributable fractions (AFs) of child admissions attributed to heat effects > 24.1 °C (Figure 4a), population-weighted annual average temperatures (Figure 4b), and the average number of days exceeding 24.1 °C (Figure 4c), all of which exhibit similar patterns. The average AF increased from 0.60% in 2013 to 1.15% in 2019, consistent with observed temperature trends.

Figure 4d shows that between 2013 and 2019, on average, there were 290 child admissions per year due to heat effects exceeding 24.1 °C. If daily maximum temperatures are to rise by 1 °C, 2 °C, and 3 °C due to climate change, these admissions are projected to increase to 510, 840, and 1300, respectively. The areas more exposed to higher temperatures (Supplemental Figure S8) are projected to be more impacted by further increases in temperature.

The spatial patterns of AFs are shown in Figure 5a. AFs were higher along the east coast of both the North and South Islands compared to the west. Some areas had particularly high AFs, including in the North Island: Waikato Region (Tuakau South 6.06%, Miranda-Pūkorokoro 3.33%), Hawke’s Bay Region (Napier Central 3.51%, Mangateretere 3.04%, Poraiti Flat 3.04%), and Gisborne Region (Makaraka-Awapuni 3.19%); and in the South Island: Canterbury Region (Aviemore 8.07%, Christchurch Airport 5.51%, Russley 3.82%, Parnassus 3.70%, Mackenzie Lakes 3.19%, Sydenham Central 3.04%), Otago Region (Clyde 4.51%, Earnscleugh 4.20%, Kingston 3.59%, Dunstan-Galloway 3.54%), and Marlborough Region (Lower Wairau 3.02%). The AFs in the remaining SA2s were below 3% (Figure 5a, interactive map in Supplemental Figure S9a).

Spatial patterns of the attributable density (AD) showed a different variation across SA2s, with a mean of 7.1 admissions per 10 km^2^ and a median of 1.8. AD was highest in the populated areas (Figure 5b). Some SA2s had particularly high ADs, including areas in the North Island: Hawke’s Bay Region (Lochain Park 130.1, Flaxmere South 126.3, Flaxmere West 122.1, Maraenui 72.5, Camberley 66.6), Waikato Region (Crawshaw 97.4, Swarbrick 94.3, Enderley North 79.6, Enderley South 75.2, Bader 63.8, Fairfield of Hamilton City 63.1, Melville North 63.0, Kahikatea 62.5), and Auckland Region (Randwick Park West 81.1, Sutton Park 72.0, Clendon Park West 68.3, Clendon Park East 62.9, Hobson Ridge Central 62.6); and in the South Island: Canterbury Region (Aranui 60.1). The remaining SA2s were below 60 per 10 km^2^ (Figure 5b, interactive map in Supplemental Figure S9b).

## 4. Discussion

We report short-term associations between ambient temperatures and daily child admissions over the past two decades in NZ. Our study applied distributed lag non-linear models (DLNMs) using a case time series study design. Given the sufficient sample size and the selection of high-quality national data, our study was able to obtain precise estimates, providing detailed insights into exposure–response relationships [23].

Our study had several strengths. We were able to access long time series of high-quality national weather and health outcome information. The ability to link administrative datasets in the data laboratory allowed us to explore the effect of individual-level factors on temperature effects. We had sufficient statistical power to demonstrate heat effects by cause of hospital admission (Supplemental Table S1). This assessment allowed us to capture a wide spectrum of potential heat-related health impacts, providing a more holistic understanding of the overall public health impact.

Statistically significant associations have been reported between higher temperatures and hospital admissions in children for diseases categorized under ICD-10 codes A, B: infectious diseases; D50-89: hematologic and immune disorders; E: endocrine, nutritional, and metabolic diseases; G: nervous system; H60-95: (ear); J: respiratory; L: skin, N: genitourinary system; R: symptoms, signs, and abnormal clinical and laboratory findings; S, T: injury, poisoning; and V: external causes of morbidity [24,26,27]. For other ICD categories, no previous studies reported the associations that we found in children, including H00-59: eye, I: cardiovascular; P: conditions originating in the perinatal period, and Z03: medical observation for suspected diseases and conditions ruled out. However, while others have found associations with ICD category K: digestive system [26], our study did not find such associations. A shorter cumulative lag structure (e.g., lag 0–1 days) might capture potential heat-related exacerbation of symptoms in diseases that are not directly related to short-term effects of heat in children, such as neoplasms (C00-D49) and congenital conditions (Q00-99) (Supplemental Figure S6).

The rationale for breaking down the ICD codes in our method lies in the need to capture the diverse ways in which heat exposure might influence health outcomes. By analysing the principal and the first two secondary causes of hospital admissions, we ensure that we account for both direct and indirect effects of heat, as these effects may manifest through multiple, sometimes less obvious, causes. Stratifying daily admission counts by broad ICD code categories helps in identifying statistical associations and assessing biological plausibility, enabling a more precise understanding of which conditions are truly heat-related. This approach allows us to refine the definition of heat-related admissions, improving the characterization of heat impacts within the healthcare system. It also ensures that our model parameters reflect a comprehensive view of the potential health risks associated with ambient heat exposure, making the results more applicable for healthcare planning and policy.

The overall lag pattern of heat effects in our study (Figure 3) aligns with findings from Australia, where both immediate and delayed heat-related health impacts were identified, with the most pronounced delayed effects occurring on day 21 [24]. Heat-related health impacts can accumulate over days or even weeks, especially among children, who are particularly vulnerable to sustained environmental stress. Initial heat exposure may not result in immediate hospitalisation, but serious illness or complications can still emerge after 2–3 weeks (Supplemental Figure S1b). These delays may be due to the incubation period of certain infectious diseases in children (up to 21 days) [28,29], delayed health-seeking behaviours of parents (up to 14 days for their children under five) [30], prolonged hypohydration effects (among >50% of children) [31], or cumulative impacts due to combinations of these or other factors.

However, it is also important to consider that some of the observed heat effects at longer lags could be due to model artefacts, especially given the complexities involved in accurately capturing long-term environmental exposures and delayed health outcomes in time series models.

Our findings align with the documented adverse health outcomes among Māori and Pacific children [32], such as increased hospitalisation rates. These children face greater hospital admission risks compared to their European and Other ethnic counterparts. The health disparities experienced by Māori and Pacific children exist within a broader context of structural disadvantages and persistent inequities across various sectors including poverty, housing, education, and justice [33]. Moreover, our study indicates that, relative to Europeans, Asian children were more vulnerable to heat effects (though less so to cold). While Asians are not typically considered the most at-risk group in terms of health disparities, the compounded effects of stress-related racial prejudice and discrimination might reduce their resilience, leading to poorer health and well-being [34], particularly under heat-stress conditions.

Deprivation, closely linked to ethnicity, lessens the material resources that individuals, families, and communities might use to mitigate the impacts of rising temperatures due to climate change or to minimise exposure [35]. The level of deprivation influences the affordability of housing in terms of quality, location, and measures to cope with heat. Our research further indicates that children in the most deprived areas were more sensitive to heat-related effects. This heightened sensitivity may be due to the ethnic concentrations in specific regions, such as Māori children in the Bay of Plenty and Pacific children in Northland and Waikato. These areas exhibit high levels of deprivation, affecting employment, income, crime rates, housing quality, health, education, and access to services [36]. Our study maps these vulnerable areas with greater detail (Supplemental Figure S9). Although these geographical and deprivation domains provide some insights, they do not fully account for the nuances in smaller areas. Further research is needed to explore ethnic-specific data with a focus on modifiable factors [35], such as health service access routes for Māori and Pacific populations and decision-making processes of parents or caregivers—particularly whether children aged 0–5 years should be taken to a hospital or primary care. Studies should also examine ethnic-specific protective factors, including multigenerational living, communal child-rearing, and co-sleeping practices [37,38].

In contrast to a previous study of heat wave effects on child hospital admissions in South Korea [14], we found that girls appeared to be more sensitive to heat than boys. Although evidence for gender disparity among children is otherwise scarce [27], it has been suggested that adult females may need more heat acclimatisation sessions than males to achieve similar physiological adaptations [39]. The underlying mechanisms are not clear, but may include hormonal factors, differences in sweat gland distribution, and variations in body composition. Females generally have a higher body fat percentage and lower skin surface area-to-mass ratio, which can affect heat dissipation and overall thermal comfort [39].

In the main models, steeper slopes at higher temperatures, compared to the flatter slopes at lower temperatures, suggest that young children were generally more sensitive to heat than to cold. Our findings suggest that non-resident children (mainly migrants) were less sensitive to heat but significantly more sensitive to cold compared to local children. Currently, no studies have reported similar associations. New Zealand receives a substantial number of migrants from lower-latitude countries, particularly India and the Philippines [40]. An acclimatisation study in Japan found that individuals from tropical regions have higher heat tolerance but are less adapted to cold environments [41]. This might explain our observations regarding migrant children’s sensitivity to temperatures; however, existing data remain insufficient to draw definitive conclusions.

We found that children living in areas with higher long-term average temperatures appeared to be more sensitive to heat. We found no other research showing similar sensitivity patterns among children. This finding might reflect the higher proportion of Māori and Pacific ethnicities in these areas. Persistent exposure to higher temperatures in the general population can lead to cumulative stress, worsen underlying health conditions, and increase mortality and morbidity rates [42].

We also show that homeowners’ children appear to be less sensitive to heat. Homeowners typically have more resources and incentives to invest in housing adaptations [43] that enhance the resilience of their homes to extreme heat, such as better ventilation and installation of shading, which can significantly reduce indoor temperatures during heat waves. Further research on heat-related effects among children will help substantiate our findings and provide a deeper understanding of the impacts of heat exposure on different demographic groups.

The attributable fraction of heat-related child admissions varied according to annual temperature patterns; in 2019 the AF was 1.15%. This highlights the importance of monitoring temperature trends and implementing effective heat mitigation strategies to protect young children in Aotearoa New Zealand.

We estimated that 290 child hospital admissions per year were attributable to heat effects. The number of admissions rises exponentially with each 1 °C increase in temperature, reaching up to 1300 admissions per year with a 3 °C increase, assuming no adaptation. This suggests that children’s vulnerability to heat stress is highly sensitive to temperature changes, consistent with existing research that indicates children are particularly susceptible to heat-related illnesses due to their developing physiological systems and higher metabolic rates [4].

The significant rise in hospital admissions with increasing temperatures underscores the urgency for public health interventions and climate adaptation strategies. These strategies should include enhancing community awareness about the dangers of heat exposure, improving access to cooling facilities, and ensuring that urban planning and housing infrastructure are designed to mitigate heat effects. Such measures are crucial to prevent the escalating burden on healthcare systems and to protect public health as global temperatures continue to rise.

We estimated the attributable fractions for finer spatial areas (SA2s) and created interactive maps (Supplemental Figure S9) to indicate localized small-area health impact estimations. These maps are useful for policymakers to identify local hot spots with higher heat-related impacts on young children. It should be noted that the map of attributable fractions (Figure 5a) does not account for the density of the vulnerable population. Some places with larger attributable fractions appeared to be associated with more days with hotter temperatures (Supplemental Figure S8), particularly in Central Otago, Hawke’s Bay, and Gisborne.

The map of attributable number density (which accounts for the density of the vulnerable population) and the map of attributable fraction serve different purposes and should be used accordingly from a policy perspective. The map of attributable fraction is best used when identifying regions with a higher relative impact of heat-related child admissions, regardless of population size. This map is useful for highlighting areas where the proportion of heat-related health impacts is highest, regardless of the rurality, allowing policymakers to prioritise interventions in regions with the greatest relative risk, raise awareness, and allocate resources for community-based prevention programmes. Conversely, the map of attributable density is more suitable for identifying regions with the highest absolute number of heat-related events. This map is crucial for understanding the total impact and planning targeted interventions, such as deploying healthcare resources, emergency services, and infrastructure improvements in areas with the greatest absolute burden of heat-related incidents. Thus, while the map of attributable fraction focuses on relative risks, the map of attributable density addresses the absolute burden, guiding effective resource allocation and intervention planning.

This study has focused only on childhood hospital admissions in the context of high temperatures. However, other studies also demonstrate clear evidence of statistically significant relationships between heat and adverse health outcomes in vulnerable older populations [44]. Future research is needed to assess this risk within a New Zealand context, particularly given that the size of the national population aged over 85 years is projected to quadruple within the next fifty years [45].

Our study had some limitations. We lacked detailed individual-level information about housing quality and socioeconomic position that might better explain the observed patterns of the heat effect. Due to limitations of computing power in the secure data laboratory, we were unable to estimate AFs for the whole study period. Uncertainty in these statistical models increases when analysing extremely high temperatures, especially those predicted beyond the range with observed hospital admissions. Despite this, these results consistently indicate elevated health risks associated with locally extreme temperatures across all regions of the country (Supplemental Figure S5). Since our study focused on the under-five population, the generalizability of our findings to older children is limited. Nevertheless, evidence suggests that children under five are more sensitive to heat than those aged 5 to 9 years old [24].

While we controlled for seasonality and long-term trends using the year and month in the model, other environmental factors, such as the El Niño–Southern Oscillation (ENSO) phenomenon, variations in humidity, and rainfall, could have an impact on how heat affects young children. The ENSO, in particular, is known to cause significant fluctuations in temperature and can lead to extreme heat or cold spells, which could amplify or mitigate the short-term effects of ambient heat on child health. These temperature anomalies may influence both the frequency and intensity of heat-related hospitalisations, as extreme weather patterns often coincide with other environmental stressors such as droughts or heavy rainfall, further exacerbating health risks. Additionally, humidity and rainfall can modify how heat is experienced by young children. High humidity, for instance, can impair the body’s ability to cool down through sweat, potentially increasing the risk of heat stress, while variations in rainfall can affect waterborne diseases, which may interact with heat exposure to influence child morbidity. The above-mentioned factors were not explicitly accounted for in our model. Further research is needed to provide a better understanding of how complex climatic factors interact with heat-related health outcomes in children.

Given New Zealand’s predominantly temperate climate, there may be limited awareness and preparedness for exposure to high temperatures, including heat waves, which could impact readiness for future trends or risks. This underestimation of heat risks was also observed in Scotland, a country with generally cooler temperatures than New Zealand, presenting unique challenges for managing heat-related health issues [46]. Child-specific temperature policies should take into account the various environments where children spend significant amounts of time, such as homes, play centres, educational institutions, local neighbourhoods, and parks, and during transport. Existing health and sectoral plans might require adjustments. For instance, while heat waves are not recognised as a national hazard in New Zealand’s Civil Defence Plan, they are acknowledged in the Heat Health Plans [47]. Regular updates to these plans are crucial for addressing emerging climate challenges and health risks, ensuring proactive measures for community safety and resilience [48]. Potential policy approaches to reduce temperature-related risks, summarised in Table 1, are based on relevant literature [49] and insights from individuals in government and non-government agencies. National adaptation plans need to include child-specific adaptation measures, acknowledging their heightened susceptibility to heat and other climate-related effects [50].

## 5. Conclusions

Our findings demonstrate significant short-term associations between local ambient temperatures and childhood hospital admissions in Aotearoa New Zealand. The temperature–response curve shows that child morbidity is sensitive to daily temperature, especially among socioeconomically disadvantaged and vulnerable ethnic groups such as Māori, Pacific, and Asian children. This heat sensitivity varies by individual- and area-level factors, indicating the importance of tailored interventions. Climate change policies should follow a “Health in All Policies” approach, encompassing the health sector, public health, and community health approaches that connect with wider determinants of health.

## Figures and Tables

**Table 1:** Opportunities to reduce heat-related health impacts to pregnant mothers, infants, and children.

Policy	Examples
a. Promoting and honouring the Treaty of Waitangi (Te Tiriti o Waitangi)	
Advancing indigenous perspectives and solutions	(1) Collaboratively design solutions, and (2) jointly implement actions that specifically cater to the needs of Māori, Pacific, and Asian children, including indigenous solutions.
b. Promoting equity and equitable solutions	
All policy promotes equity and equitable solutions	(1) Respect and uphold the principles of absolute sovereignty and self-determination (tino rangatiratanga), and allocate funds to support kaupapa approaches. (2) The most vulnerable and at-risk children are prioritised.
c. Knowing the facts: climate change is increasing temperatures in New Zealand and children are increasingly exposed	
Knowledge transfer	(1) Share research findings and create opportunities to share knowledge on child-related health impacts, prevention, and adaptation to heat, (2) with a focus on pregnant women and infants and children.
Mainstreaming existing policies	(2) Craft policy briefs to effectively communicate research findings and facilitate their widespread distribution. (3) Incorporate temperature and health impacts into pertinent policy documents for comprehensive consideration. (4) Integrate and recognise synergies with existing climate adaptation plans, such as the Health National Adaptation Plan. (5) Develop national guidelines for educational initiatives addressing maximum temperatures to safeguard health and ensure safety. (6) Encompass climate mitigation and strategies for coping with extreme temperatures within Long-Term Plans. (7) Embrace a “Health in All Policies” approach, fostering a comprehensive consideration of health implications across all policy domains.
d. Focus on prevention	
Risk communication and surveillance	(1) Pregnant mothers, parents, carers, and those working with children have the knowledge to reduce the risk of heat-related impacts.
Enhancing governance in child heat health	(2) Foster cross-sectoral collaboration at both local and national levels, forging connections with established experts, such as the National Emergency Management Agency’s heat health group, MetService, the Ministry for the Environment, and the Ministry of Health. (3) Ensure adequate resourcing to effectively implement policy approaches. (4) Comply with and leverage existing legislation to strengthen the foundation of the initiatives and enhance their effectiveness.
e. Treatment in healthcare facilities	
Review healthcare practices for pregnant women, infants, and children	(1) Enable diagnosis of heat-related illness in clinical settings. (2) Establish case management flows for diagnosing and treating heat-related illness in vulnerable populations. (3) Understand medications, complications, and comorbidities that relate to heat stress. (4) Understand comorbidities and the impact of health emergencies.
f. Invest in preparedness	
Preparing child-specific and Early Childhood Education (ECE) centres	(1) Incorporate critical considerations into health and safety assessments and ensure compliance, including the provision of adequate shade, ventilation, water, and cooling/air conditioning. (2) Provide support for non-governmental organizations (NGOs) and independent groups, such as play centres, to enhance their capacity and effectiveness in implementing these considerations.
Preparing healthcare facilities	(3) Implement strategies for shade provision, cooling methods, emissions reduction, and adequate access to water resources. (4) Develop and enact Regional Heat Health Plans to address localized challenges and ensure comprehensive heat management strategies.
Planning and implementation of risk communication and outreach campaigns for educational or behavioural change	(5) Develop targeted public health messaging with tailored advice for parents and child caregivers. (6) Launch campaigns aimed at raising public awareness and delivering essential services for effective heat wave management. (7) Ensure consistent messaging that draws connections between addressing health impacts associated with both hot and cold temperatures, as well as air quality concerns. (8) Implement early warning systems that are integrated into healthcare planning and ECE settings for a proactive response.
g. Promote national action: heat adaptation measures that can be implemented by national governments	
Advancing housing standards and guidelines	(1) Introduce new standards, codes, action plans, or infrastructural upgrades for buildings to enhance thermal comfort, keeping homes cool in summer and warm in winter. (2) Ensure rental houses adhere to guidelines for mitigating heat and cold, such as the Rental Warrant of Fitness and World Health Organization housing and health recommendations.
Addressing the interplay between housing and socioeconomic factors	(3) Provide support for vulnerable populations to prevent heating/cooling poverty, including initiatives like the Healthy Homes initiative, and (4) the expansion of grants from the Energy Efficiency and Conservation Authority or the Ministry for Business, Innovation and Employment. (5) Enhance safety in neighbourhoods and buildings through improved security features, facilitating natural ventilation.
h. Promote local action: heat adaptation measures that can be implemented by local or regional governments	
Navigating the intersection of housing and urban planning in local neighbourhoods	(1) Mitigate the urban heat island effect by incorporating strategic tree provision, recognising the necessary time for trees to mature, especially in new housing developments. (2) Enhance access to green and blue spaces, addressing disparities in tree canopy coverage. (3) Install drinking water fountains in schools, playgrounds, and other public areas. (4) Implement shading in parks and playgrounds to create comfortable outdoor spaces. (5) Strengthen the resilience of the water supply and critical infrastructure to manage increased demand during heat waves. (6) Provide shade and shelter in transportation settings. (7) Establish cooling centres, utilizing recreation facilities and other public spaces like shopping malls, and promote active health opportunities, such as swimming and indoor sports.
Promoting green and natural infrastructure	(8) Implement nature-based greening and water solutions to promote sustainable and environmentally friendly practices.
i. Promote a multisectoral national response plan	
Developing a Health National Adaptation Plan with a focus on children and other vulnerable populations	(1) Provide effective co-ordination mechanisms to protect human health from excess heat. (2) Provide effective polices for coping with excess heat. (3) Provide early warning and notification systems. (4) Ensure a primary healthcare approach to protecting children and other vulnerable populations from heat stress. (5) Adapt built environments for the long term (see g.1 to g.5 and h.1 to h.3).
Conducting comprehensive research and evaluation	(6) Draw insights from adaptation examples in countries with extensive experience in managing heat health, such as Australia. (7) Foster connections and effective communication channels for disseminating heat-related research to the appropriate end users. (8) Evaluate the return on investment of implemented policies to better understand their effectiveness and impact.

## Data Availability

De-identified hospital admission data and temperature data are publicly available from Stats NZ (access2microdata@stats.govt.nz) and NIWA (enquiries@niwa.co.nz), respectively. Model output data on temperature-specific RR (as shown in the graphs) can be shared with researchers who request access to the data. Requests should be sent to the corresponding author.

## Supplementary Materials

The following supporting information can be downloaded at https://www.mdpi.com/article/10.3390/ijerph21091236/s1, Figure S1: Sensitivity analysis—temperature–response curves (cumulative lags) for child admissions in 2000–2019—varied by lag range.; Figure S2: Sensitivity analysis—temperature–response curves (cumulative lag 0–21 days) for child admissions in 2000–2019—varied by knots; Figure S3: Methods to derive the housing tenure from the 2013 census data; Figure S4: Sensitivity analysis—temperature–response curves (cumulative lag 0–21 days) for child admissions—stratified by study periods; Figure S5: Sensitivity analysis—temperature–response curves (cumulative lag 0–21 days) for child admissions—modified by 16 Regional Council areas of New Zealand; Figure S6: Three-dimensional plots by ICD groups; Figure S7: No effect modification by proportion of private rental households; Figure S8: Spatial variation in average daily maximum temperatures and the population’s exposure to heat in New Zealand in 2019; Figure S9: Interactive maps: child hospital admissions due to heat effects > 24.1 °C (cumulative lag 0–21 days) from 2013–2019—spatial patterns of attributable fractions (%) and attributable density (number per 10 km^2^) at statistical area (SA2) level; Table S1: Summary of inclusion and exclusion of ICD codes to define heat-related child admissions between 2000 and 2019 for the analyses of exposure–response (cumulative lag 0–21 days) patterns in this study; Video S1: Slide show of the paper (~12 min).

## References

[1] Costello A., Abbas M., Allen A., Ball S., Bell S., Bellamy R., Friel S., Groce N., Johnson A., Kett M. (2009). Managing the health effects of climate change: Lancet and University College London Institute for Global Health Commission. Lancet.

[2] Romanello M., Napoli C.D., Green C., Kennard H., Lampard P., Scamman D., Walawender M., Ali Z., Ameli N., Ayeb-Karlsson S. (2023). The 2023 report of the Lancet Countdown on health and climate change: The imperative for a health-centred response in a world facing irreversible harms. Lancet.

[3] Rogelj J., Fransen T., den Elzen M.G.J., Lamboll R.D., Schumer C., Kuramochi T., Hans F., Mooldijk S., Portugal-Pereira J. (2023). Credibility gap in net-zero climate targets leaves world at high risk. Science.

[4] Xu Z., Sheffield P.E., Su H., Wang X., Bi Y., Tong S. (2014). The impact of heat waves on children’s health: A systematic review. Int. J. Biometeorol..

[5] Philipsborn R.P., Chan K. (2018). Climate Change and Global Child Health. Pediatrics.

[6] Sheffield P.E., Landrigan P.J. (2011). Global Climate Change and Children’s Health: Threats and Strategies for Prevention. Environ. Health Perspect..

[7] Sheffield P.E., Weinberger K.R., Kinney P.L. (2011). Climate change, aeroallergens, and pediatric allergic disease. Mt. Sinai J. Med..

[8] Clarke B., Otto F., Stuart-Smith R., Harrington L. (2022). Extreme weather impacts of climate change: An attribution perspective. Environ. Res. Clim..

[9] Vicedo-Cabrera A.M., Scovronick N., Sera F., Royé D., Schneider R., Tobias A., Astrom C., Guo Y., Honda Y., Hondula D.M. (2021). The burden of heat-related mortality attributable to recent human-induced climate change. Nat. Clim. Chang..

[10] Ebi K.L. (2022). Managing climate change risks is imperative for human health. Nat. Rev. Nephrol..

[11] Lakhoo D.P., Blake H.A., Chersich M.F., Nakstad B., Kovats S. (2022). The Effect of High and Low Ambient Temperature on Infant Health: A Systematic Review. Int. J. Environ. Res. Public Health.

[12] Chersich M.F., Pham M.D., Areal A., Haghighi M.M., Manyuchi A., Swift C.P., Wernecke B., Robinson M., Hetem R., Boeckmann M. (2020). Associations between high temperatures in pregnancy and risk of preterm birth, low birth weight, and stillbirths: Systematic review and meta-analysis. BMJ (Clin. Res. Ed.).

[13] Iñiguez C., Schifano P., Asta F., Michelozzi P., Vicedo-Cabrera A., Ballester F. (2016). Temperature in summer and children’s hospitalizations in two Mediterranean cities. Environ. Res..

[14] Oh J., Kim E., Kwag Y., An H., Kim H.S., Shah S., Lee J.H., Ha E. (2024). Heat wave exposure and increased heat-related hospitalizations in young children in South Korea: A time-series study. Environ. Res..

[15] Neff J.M., Clifton H., Park K.J., Goldenberg C., Popalisky J., Stout J.W., Danielson B.S. (2010). Identifying children with lifelong chronic conditions for care coordination by using hospital discharge data. Acad. Pediatr..

[16] McMichael A.J., Wilkinson P., Kovats R.S., Pattenden S., Hajat S., Armstrong B., Vajanapoom N., Niciu E.M., Mahomed H., Kingkeow C. (2008). International study of temperature, heat and urban mortality: The ‘ISOTHURM’ project. Int. J. Epidemiol..

[17] Zhao Q., Guo Y., Ye T., Gasparrini A., Tong S., Overcenco A., Urban A., Schneider A., Entezari A., Vicedo-Cabrera A.M. (2021). Global, regional, and national burden of mortality associated with non-optimal ambient temperatures from 2000 to 2019: A three-stage modelling study. Lancet Planet Health.

[18] Gasparrini A., Guo Y., Hashizume M., Lavigne E., Zanobetti A., Schwartz J., Tobias A., Tong S., Rocklov J., Forsberg B. (2015). Mortality risk attributable to high and low ambient temperature: A multicountry observational study. Lancet.

[19] Cure Kids, New Zealand Child & Youth Epidemiology Service, Paediatric Society of New Zealand, Royal Australasian College of Physicians (2023). State of child health in Aotearoa New Zealand 2022.

[20] MoH (2002). Reducing Inequalities in Health.

[21] Young P.C., Korgenski K., Buchi K.F. (2013). Early readmission of newborns in a large health care system. Pediatrics.

[22] NIWA Climate Data—NIWA Maintains the National Climate Database for New Zealand, and Can Supply Climate Data in a Variety of Ways. https://niwa.co.nz/climate-and-weather/climate-data.

[23] Gasparrini A., Masselot P., Scortichini M., Schneider R., Mistry M.N., Sera F., Macintyre H.L., Phalkey R., Vicedo-Cabrera A.M. (2022). Small-area assessment of temperature-related mortality risks in England and Wales: A case time series analysis. Lancet Planet Health.

[24] Xu Z., Hu W., Su H., Turner L.R., Ye X., Wang J., Tong S. (2014). Extreme temperatures and paediatric emergency department admissions. J. Epidemiol. Community Health.

[25] Gasparrini A., Leone M. (2014). Attributable risk from distributed lag models. BMC Med. Res. Methodol..

[26] Bernstein A.S., Sun S., Weinberger K.R., Spangler K.R., Sheffield P.E., Wellenius G.A. (2022). Warm Season and Emergency Department Visits to U.S. Children’s Hospitals. Environ. Health Perspect..

[27] Uibel D., Sharma R., Piontkowski D., Sheffield P.E., Clougherty J.E. (2022). Association of ambient extreme heat with pediatric morbidity: A scoping review. Int. J. Biometeorol..

[28] Czumbel I., Quinten C., Lopalco P., Semenza J.C., Tozzi A.E., Weil-Oliver C., Nichols G., Nøkleby H., Brumboiu I., Verheijen J. (2018). Management and control of communicable diseases in schools and other child care settings: Systematic review on the incubation period and period of infectiousness. BMC Infect. Dis..

[29] Huang N.N., Zheng H., Li B., Fei G.Q., Ding Z., Wang J.J., Li X.B. (2021). The Short-term Effects of Temperature on Infectious Diarrhea among Children under 5 Years Old in Jiangsu, China: A Time-series Study (2015–2019). Curr. Med. Sci..

[30] Pajuelo M.J., Anticona Huaynate C., Correa M., Mayta Malpartida H., Ramal Asayag C., Seminario J.R., Gilman R.H., Murphy L., Oberhelman R.A., Paz-Soldan V.A. (2018). Delays in seeking and receiving health care services for pneumonia in children under five in the Peruvian Amazon: A mixed-methods study on caregivers’ perceptions. BMC Health Serv. Res..

[31] Liska D., Mah E., Brisbois T., Barrios P.L., Baker L.B., Spriet L.L. (2019). Narrative Review of Hydration and Selected Health Outcomes in the General Population. Nutrients.

[32] Duncanson M., Roy M., van Asten H., Oben G., Wicken A., Tustin K., McAnally H., Adams J. (2022). Child Poverty Monitor 2022 Technical Report.

[33] Sinclair O., Lyndon M. (2023). Pathways towards Health Equity for Tamariki MĀORI.

[34] Liu L.S., Jia X., Zhu A., Ran G.J., Siegert R., French N., Johnston D. (2023). Stigmatising and Racialising COVID-19: Asian People’s Experience in New Zealand. J. Racial Ethn. Health Disparities.

[35] Hansen A., Bi L., Saniotis A., Nitschke M. (2013). Vulnerability to extreme heat and climate change: Is ethnicity a factor?. Glob. Health Action.

[36] Exeter D.J., Zhao J., Crengle S., Lee A., Browne M. (2017). The New Zealand Indices of Multiple Deprivation (IMD): A new suite of indicators for social and health research in Aotearoa, New Zealand. PLoS ONE.

[37] George M., Richards R., Watson B., Lucas A., Fitzgerald R., Taylor R., Galland B. (2021). Pacific families navigating responsiveness and children’s sleep in Aotearoa New Zealand. Sleep Med. X.

[38] Poland M., Paterson J., Carter S., Gao W., Perese L., Stillman S. (2007). Pacific Islands Families Study: Factors associated with living in extended families one year on from the birth of a child. Kōtuitui N. Z. J. Soc. Sci. Online.

[39] Wickham K.A., Wallace P.J., Cheung S.S. (2021). Sex differences in the physiological adaptations to heat acclimation: A state-of-the-art review. Eur. J. Appl. Physiol..

[40] Statistics New Zealand International Migration: January 2024. https://www.stats.govt.nz/information-releases/international-migration-january-2024.

[41] Tochihara Y., Wakabayashi H., Lee J.-Y., Wijayanto T., Hashiguchi N., Saat M. (2022). How humans adapt to hot climates learned from the recent research on tropical indigenes. J. Physiol. Anthropol..

[42] Kenny G.P., Notley S.R., Flouris A.D., Grundstein A., Adams W.M., Jardine J.F. (2020). Climate Change and Heat Exposure: Impact on Health in Occupational and General Populations. Exertional Heat Illness: A Clinical and Evidence-Based Guide.

[43] Hu M., Lin Z., Liu Y. (2024). Housing Disparity between Homeowners and Renters: Evidence from China. J. Real Estate Financ. Econ..

[44] Ballester J., Quijal-Zamorano M., Méndez Turrubiates R.F., Pegenaute F., Herrmann F.R., Robine J.M., Basagaña X., Tonne C., Antó J.M., Achebak H. (2023). Heat-related mortality in Europe during the summer of 2022. Nat. Med..

[45] Statistics New Zealand National Population Projections: 2020(base)–2073. December 2020. https://www.stats.govt.nz/information-releases/national-population-projections-2020base2073.

[46] Wan K., Lane M., Feng Z. (2023). Heat-health governance in a cool nation: A case study of Scotland. Environ. Sci. Policy.

[47] Health New Zealand Heat Health Plans: Guidelines and Key Information. September 2023. https://www.tewhatuora.govt.nz/publications/heat-health-plans-guidelines.

[48] Pourzand F., Bolton A., Salter C., Hales S., Woodward A. (2023). Health and climate change: Adaptation policy in Aotearoa New Zealand. Lancet Reg. Health West. Pac..

[49] UNICEF (2023). Protecting Children from Heat Stress—A Technical Note.

[50] Zangerl K.E., Hoernke K., Andreas M., Dalglish S.L., Kelman I., Nilsson M., Rockloev J., Bärnighausen T., McMahon S.A. (2024). Child health prioritisation in national adaptation policies on climate change: A policy document analysis across 160 countries. Lancet Child Adolesc. Health.

