# Supplementary material for: Daily Temperatures and Child Hospital Admissions in Aotearoa New Zealand: Case Time Series Analysis
Source: Int J Environ Res Public Health. 2024 Sep 19;21(9):1236. doi: 10.3390/ijerph21091236 (PMC11432253; doi:10.3390/ijerph21091236)
Supplement: Supplementary file 1 [file ijerph-21-01236-s001.zip › FigS1-S9 TableS1_Lai et al 2024_suppl.pdf]

International Journal of
Environmental Research
and Public Health

MDPI

Daily temperatures and child hospital admissions in Aotearoa New Zealand: case time series analysis

Supplemental Figure S1. Sensitivity analysis - temperature-response curves for child admissions in 2000-2019 - varied by lag range.

a. Cumulative-lag patterns

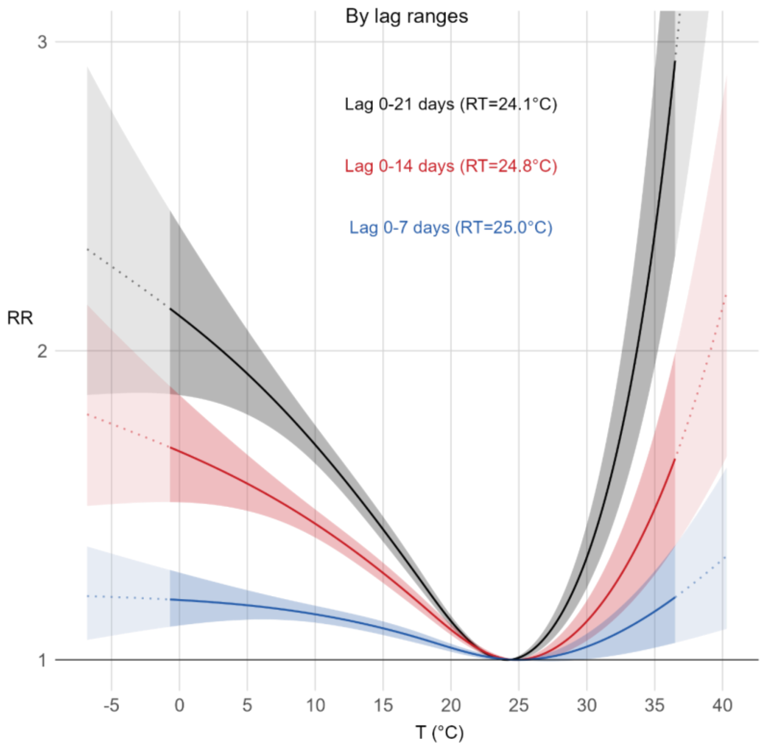

X-axis: daily maximum temperature (°C); Y-axis: relative risks (RR) of admissions; 95% CI are shaded. Model predicted RR & 95% CI beyond observable temperature range for admissions are in dotted line and lighter shade.

b. Exposure-lag-response 3D patterns

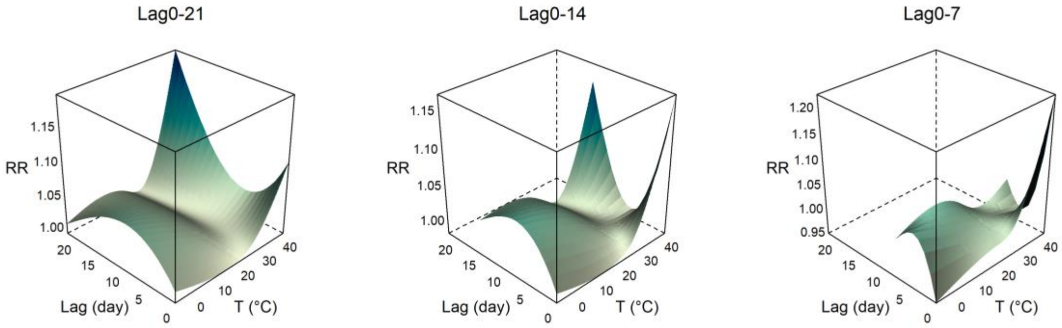

Supplemental Figure S2. Sensitivity analysis - temperature-response curves (cumulative lag 0-21 days) for child admissions in 2000-2019 - varied by knots.

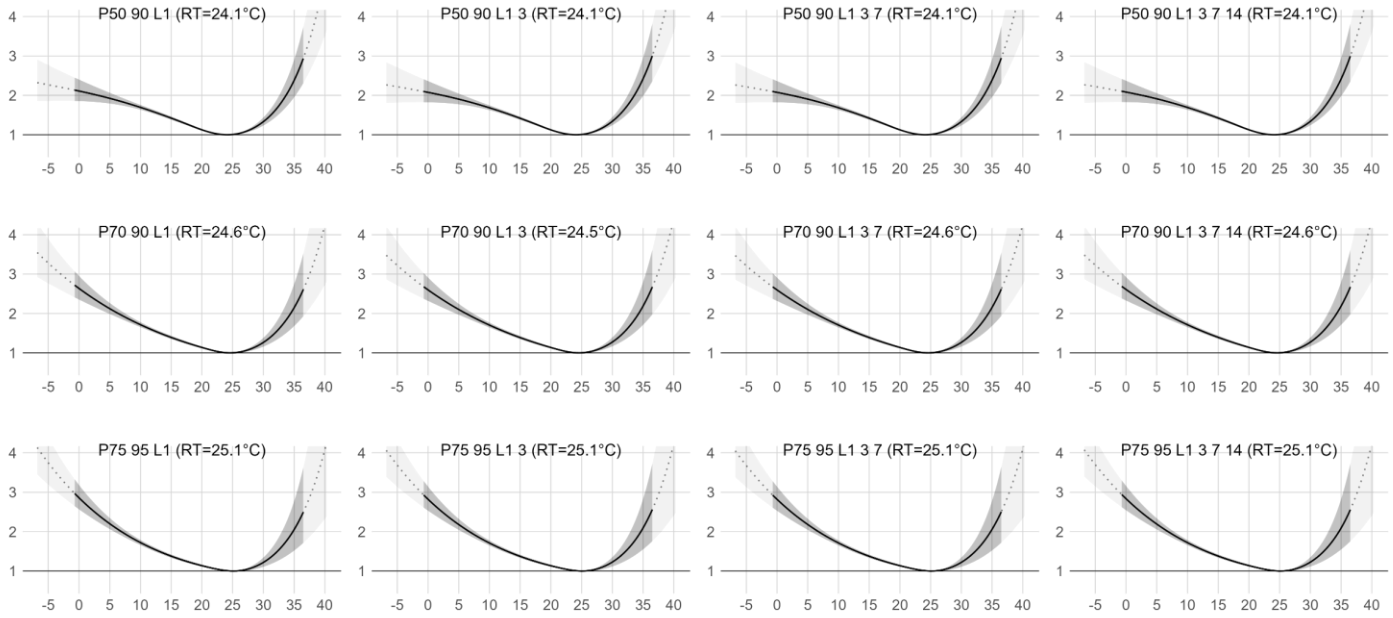

X-axis: daily maximum temperature (°C); Y-axis: relative risks (RR) of admissions; 95% CI are shaded. Model predicted RR & 95% CI beyond observable temperature range for admissions are in dotted line and lighter shade. Plots from left to right, knots were placed at lag 1, lag 1 & 3, lag 1 & 3 & 7, and lag 1 & 3 & 7 & 14, respectively. Plots from top to bottom, knots were placed at temperature percentiles of 50 & 90, 70 & 90, and 75 & 95, respectively.

Supplemental Figure S3. Methods to derive the housing tenure from the 2013 census data

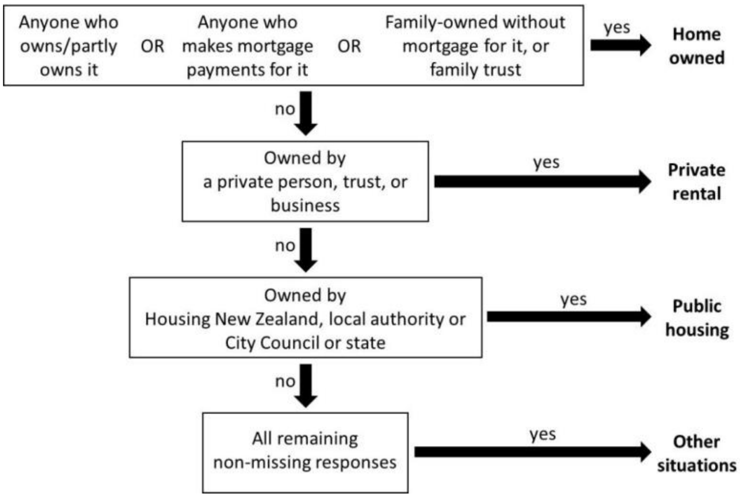

(method adopted from the Growing Up in New Zealand child cohort study)

Supplemental Figure S4. Sensitivity analysis - temperature-response curves (cumulative lag 0-21 days) for child admissions - stratified by study periods.

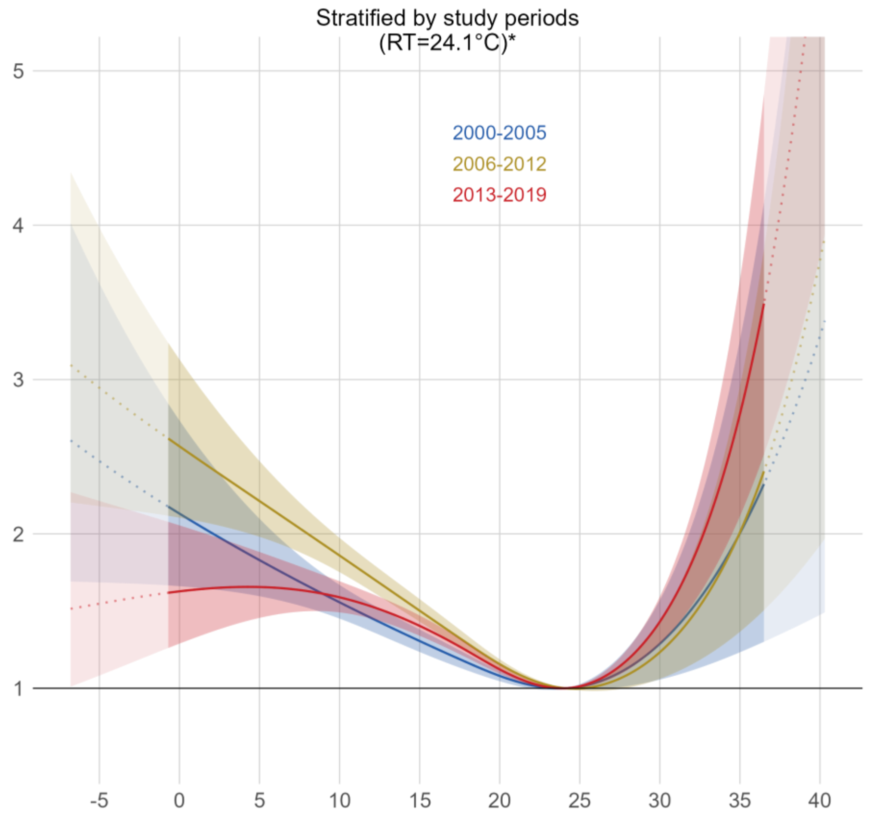

X-axis: daily maximum temperature (°C); Y-axis: relative risks (RR) of admissions; 95% CI are shaded. Model predicted RR & 95% CI beyond observable temperature range for admissions are in dotted line and lighter shade. The same reference temperature (RT), 24.1°C, were used for the three periods in this plot. Minimised RR for periods 2000-2005, 2006-2012, and 2013-2019 were found at 23.8°C, 24.7°C, and 23.9°C, respectively.

Supplemental Figure S5. Sensitivity analysis - temperature-response curves (cumulative lag 0-21 days) for child admissions - modified by 16 Regional Council areas of New Zealand.

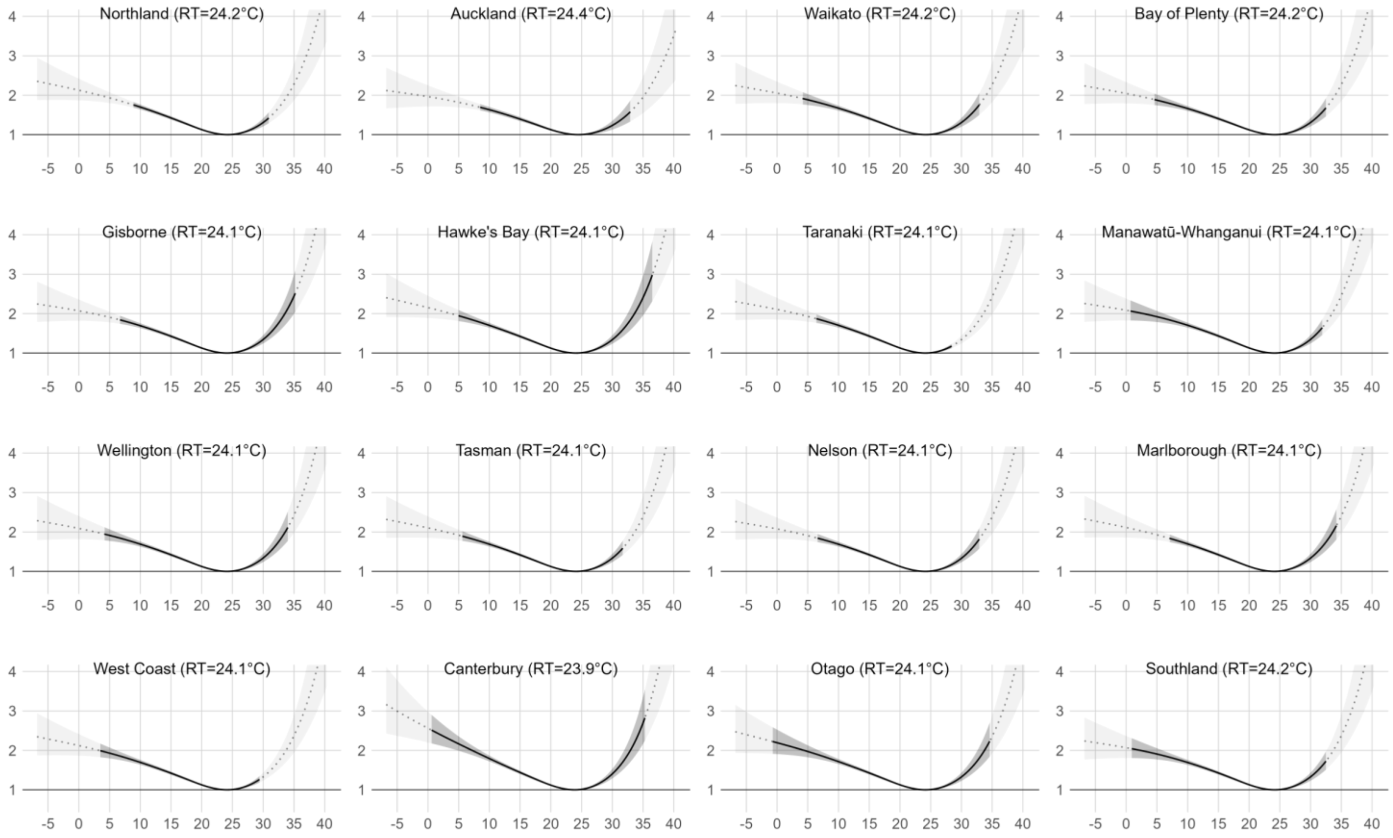

X-axis: daily maximum temperature (°C); Y-axis: relative risks (RR) of admissions; 95% CI are shaded. Model predicted RR & 95% CI beyond observable temperature range for admissions are in dotted line and lighter shade.

Supplemental Figure S6. Three-dimensional plots by ICD groups

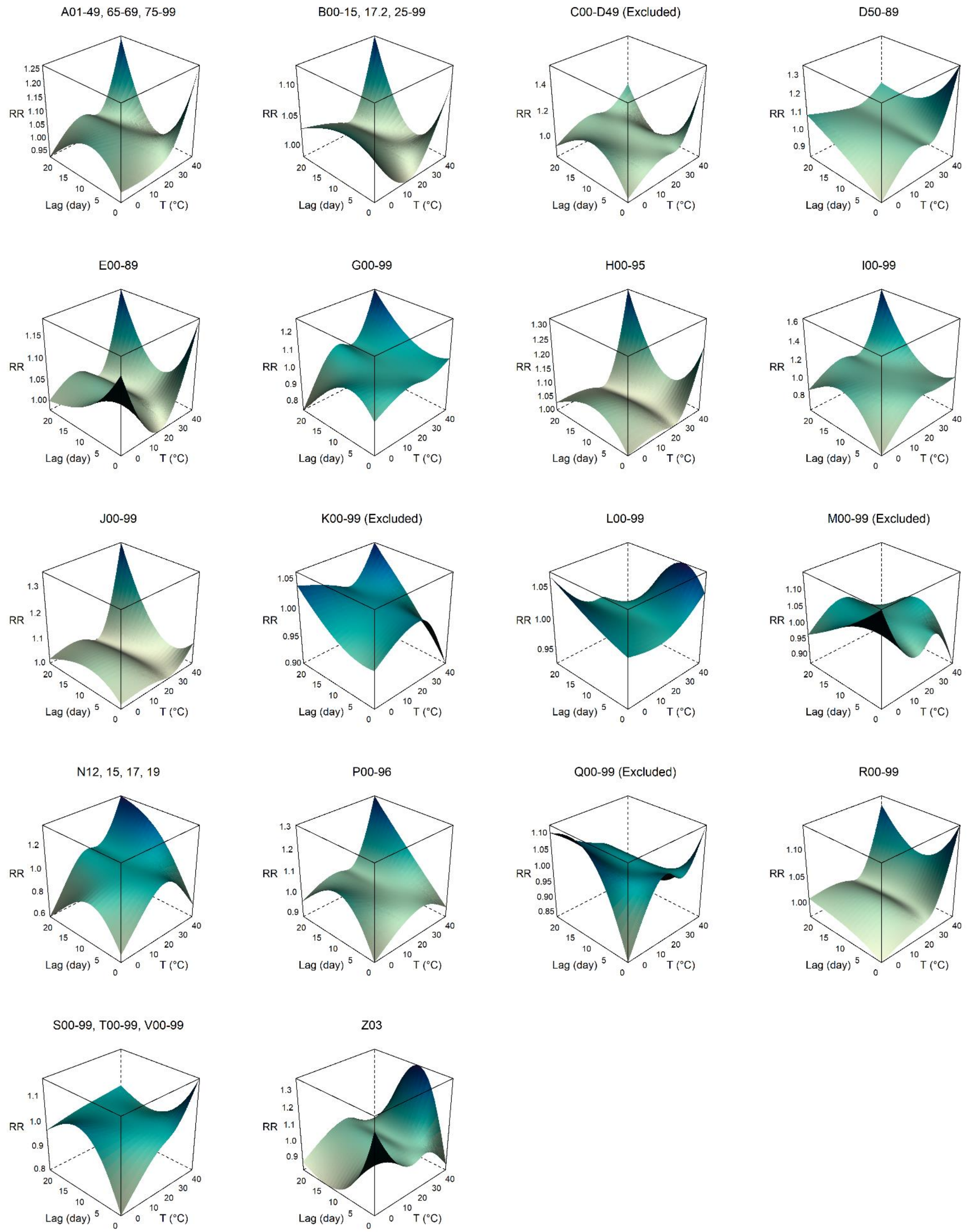

Supplemental Figure S7. No significant effect modification by proportion of private rental households

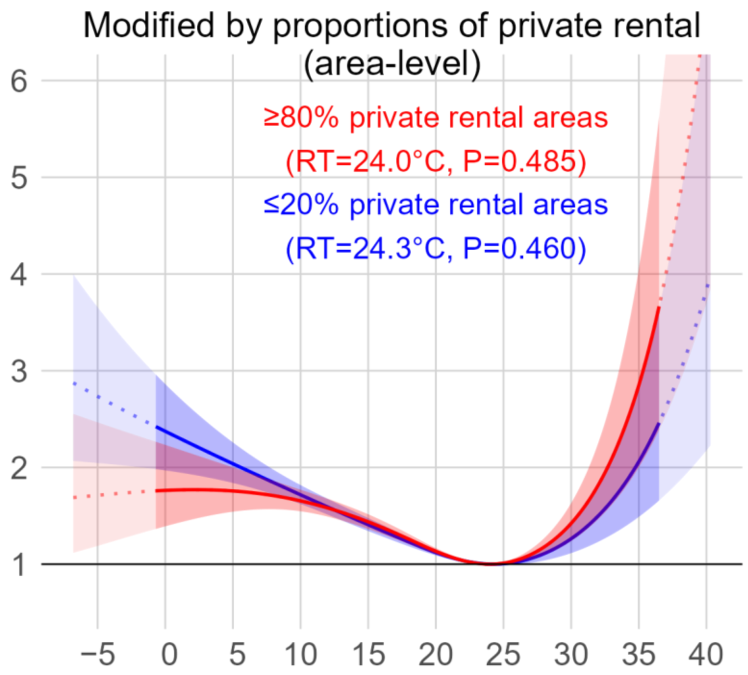

Supplemental Figure S8. Spatial variation in average daily maximum temperatures and the population's exposure to heat in New Zealand in 2019

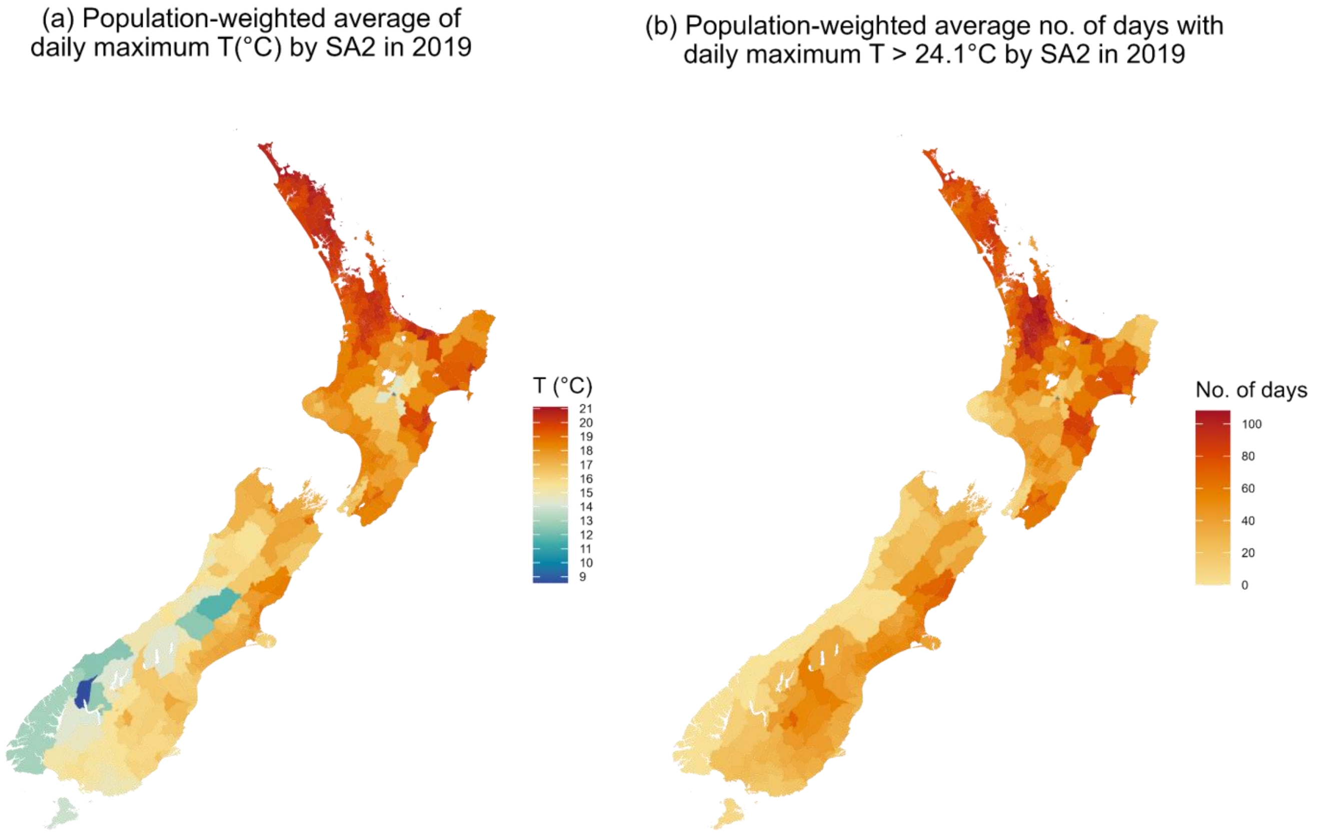

SA2-level data in (a) and (b) were aggregated from meshblock-level data

Supplemental Figure S9. Interactive maps: child hospital admissions due to heat effects >24.1°C (cumulative lag 0-21 days) from 2013-2019 – spatial patterns of attributable fractions (%) and attributable density (number per 10 km^2) at statistical area (SA2) level.

(a) Interactive map of attributable fractions

S9_Interactive_map_AF.html <- double click this icon

(b) Interactive map of attributable density

S9_Interactive_map_AD.html <- double click this icon

Supplemental Table S1. Summary of inclusion and exclusion of ICD codes to define heat-related child admissions between 2000 and 2019 for the analyses of exposure-response (cumulative lag 0-21 days) patterns in this study.

Included ICD codes		Excluded ICD codes and rationale	RT (°C)	RR (95% CI) at specific daily maximum temperatures		
**Codes**	**Descriptions**			**21°C**	**27°C**	**30°C**
A01-49, 65-69, 75-99	Bacterial, viral and other specific infectious diseases	A50-64, 70-74. E.g. Chlamydia. Mainly by sexual transmission.	24.7	1.092 (1.070-1.114)	1.039 (1.006-1.073)	1.207 (1.090-1.335)
B00-15, 17.2, 25-99	Viral, fungal and other specific infectious diseases	B16-19, B20-24. Hepatitis B, C, and HIV. Mainly by sexual transmission.	23.2	1.016 (1.005-1.027)	1.058 (1.018-1.099)	1.186 (1.073-1.312)
		C00-D49. Neoplasms. No heat effects.	24.1	0.986 (0.971-1.002)	0.976 (0.943-1.009)	0.916 (0.831-1.009)
D50-89	Haematologic and immune disorders		23.7	1.069 (1.054-1.085)	1.149 (1.105-1.195)	1.610 (1.445-1.794)
E00-89	Endocrine, nutritional and metabolic diseases		24.5	1.121 (1.098-1.144)	1.068 (1.030-1.109)	1.347 (1.205-1.506)
		F00-99. Mental and behavioural. Lack of data quality among young children.	n.a.	n.a.	n.a.	n.a.
G00-99	Nervous system diseases		23.5	1.043 (1.029-1.057)	1.126 (1.083-1.172)	1.480 (1.330-1.646)
H00-95	Eye and ear diseases		24.2	1.149 (1.126-1.172)	1.133 (1.086-1.183)	1.650 (1.460-1.865)
I00-99	Circulatory system diseases		24.0	1.085 (1.068-1.102)	1.115 (1.076-1.155)	1.503 (1.359-1.663)
J00-99	Respiratory system diseases		24.9	1.234 (1.209-1.260)	1.064 (1.034-1.096)	1.419 (1.291-1.558)
		K00-99. Digestive system. No heat effects.	24.1	1.022 (1.006-1.039)	0.961 (0.927-0.996)	0.907 (0.819-1.005)
L00-99	Skin and subcutaneous tissue diseases		18.7	1.008 (0.994-1.022)	1.103 (1.055-1.152)	1.191 (1.079-1.314)
		M00-99. Musculoskeletal and connective tissue. No heat effects.	24.1	1.022 (1.005-1.039)	0.951 (0.918-0.986)	0.876 (0.791-0.971)
N12, 15, 17-19	Renal disorders	N00-11, 13-14, 16, 20-99. Immune, infectious, drugs, toxins, anatomical, kidney stone, and genitourinary.	23.9	1.051 (1.035-1.068)	1.106 (1.064-1.149)	1.439 (1.293-1.602)
P00-96	Conditions originating in the perinatal period		21.9	1.004 (0.999-1.009)	1.224 (1.170-1.279)	1.663 (1.491-1.856)
		Q00-99. Congenital and chromosomal disorders. No heat effects.	24.1	1.103 (1.085-1.121)	0.854 (0.823-0.886)	0.679 (0.611-0.755)
R00-99	Symptoms, signs and abnormal findings		23.5	1.042 (1.029-1.055)	1.118 (1.078-1.160)	1.441 (1.305-1.592)
S00-99, T00-98, V00-99	Injuries and external causes		24.1	0.952 (0.937-0.966)	1.046 (1.014-1.080)	1.094 (0.999-1.199)
Z03	Observations for suspected conditions		23.6	1.059 (1.044-1.074)	1.159 (1.113-1.207)	1.638 (1.468-1.828)

Main model's reference temperature (RT) of 24.1°C, as underlined, was used when minimum relative risk (RR) was not observed. No data available for ICD categories O, X and Y in our cohort.
